# Guidelines for Neuroprognostication in Critically Ill Adults with Intracerebral Hemorrhage

**DOI:** 10.1007/s12028-023-01854-7

**Published:** 2023-11-03

**Authors:** David Y. Hwang, Keri S. Kim, Susanne Muehlschlegel, Katja E. Wartenberg, Venkatakrishna Rajajee, Sheila A. Alexander, Katharina M. Busl, Claire J. Creutzfeldt, Gabriel V. Fontaine, Sara E. Hocker, Dominik Madzar, Dea Mahanes, Shraddha Mainali, Oliver W. Sakowitz, Panayiotis N. Varelas, Christian Weimar, Thomas Westermaier, Jürgen Meixensberger

**Affiliations:** 1 Division of Neurocritical Care, Department of Neurology, University of North Carolina School of Medicine, 170 Manning Drive, CB# 7025, Chapel Hill, NC 27599-7025 USA; 2 Department of Pharmacy Practice, University of Illinois at Chicago College of Pharmacy, Chicago, IL USA; 3 Division of Neurosciences Critical Care, Departments of Neurology and Anesthesiology/Critical Care Medicine, Johns Hopkins Medicine, Baltimore, MD USA; 4 Department of Neurology, University of Leipzig, Leipzig, Germany; 5 Departments of Neurology and Neurosurgery, University of Michigan, Ann Arbor, MI USA; 6 School of Nursing, University of Pittsburgh, Pittsburgh, PA USA; 7 Departments of Neurology and Neurosurgery, College of Medicine, University of Florida, Gainesville, FL USA; 8 Department of Neurology, University of Washington, Seattle, WA USA; 9 Departments of Pharmacy and Neurosciences, Intermountain Health, Salt Lake City, UT USA; 10 Department of Neurology, Mayo Clinic, Rochester, MN USA; 11 Department of Neurology, University of Erlangen-Nuremberg, Erlangen, Germany; 12 Departments of Neurology and Neurosurgery, UVA Health, Charlottesville, VA USA; 13 Department of Neurology, Virginia Commonwealth University, Richmond, VA USA; 14 Department of Neurosurgery, Neurosurgery Center Ludwigsburg-Heilbronn, Ludwigsburg, Germany; 15 Department of Neurology, Albany Medical College, Albany, NY USA; 16 Institute of Medical Informatics, Biometry and Epidemiology, University Hospital Essen, Essen, Germany; 17 BDH-Klinik Elzach, Elzach, Germany; 18 Department of Neurosurgery, Helios Amper-Kliniken Dachau, University of Wuerzburg, Würzburg, Germany; 19 Department of Neurosurgery, University of Leipzig, Leipzig, Germany

**Keywords:** Hemorrhagic stroke, Cerebral hemorrhage, Prognosis, Patient outcome assessment, Critical care outcomes, Mortality, Counseling, Shared decision making, Practice guideline

## Abstract

**Background:**

The objective of this document is to provide recommendations on the formal reliability of major clinical predictors often associated with intracerebral hemorrhage (ICH) neuroprognostication.

**Methods:**

A narrative systematic review was completed using the Grading of Recommendations Assessment, Development, and Evaluation methodology and the Population, Intervention, Comparator, Outcome, Timing, Setting questions. Predictors, which included both individual clinical variables and prediction models, were selected based on clinical relevance and attention in the literature. Following construction of the evidence profile and summary of findings, recommendations were based on Grading of Recommendations Assessment, Development, and Evaluation criteria. Good practice statements addressed essential principles of neuroprognostication that could not be framed in the Population, Intervention, Comparator, Outcome, Timing, Setting format.

**Results:**

Six candidate clinical variables and two clinical grading scales (the original ICH score and maximally treated ICH score) were selected for recommendation creation. A total of 347 articles out of 10,751 articles screened met our eligibility criteria. Consensus statements of good practice included deferring neuroprognostication—aside from the most clinically devastated patients—for at least the first 48–72 h of intensive care unit admission; understanding what outcomes would have been most valued by the patient; and counseling of patients and surrogates whose ultimate neurological recovery may occur over a variable period of time. Although many clinical variables and grading scales are associated with ICH poor outcome, no clinical variable alone or sole clinical grading scale was suggested by the panel as currently being reliable by itself for use in counseling patients with ICH and their surrogates, regarding functional outcome at 3 months and beyond or 30-day mortality.

**Conclusions:**

These guidelines provide recommendations on the formal reliability of predictors of poor outcome in the context of counseling patients with ICH and surrogates and suggest broad principles of neuroprognostication. Clinicians formulating their judgments of prognosis for patients with ICH should avoid anchoring bias based solely on any one clinical variable or published clinical grading scale.

**Supplementary Information:**

The online version contains supplementary material available at 10.1007/s12028-023-01854-7.

## Introduction

Stroke is the second leading cause of death and disability worldwide, with intracerebral hemorrhage (ICH) accounting for approximately 10% of all strokes [1] and having an incidence of 24.6 per 100,000 person-years [1, 2]. Although the worldwide incidence of ICH and its associated morbidity and mortality have remained stable or decreased since the 1970s [2, 3], definitive therapeutic options remain uncertain at this time, and there is evidence that in the United States, incidence rates have actually been rising [4]. In an absolute sense, ICH still remains associated with high mortality and poor functional outcome [5, 6].

Many independent risk factors associated with high morbidity and/or mortality among patients with ICH have been identified in the literature [7, 8]. Combinations of these risk factors and clinical variables have been incorporated into dozens of ICH clinical grading scales over the past few decades [9–11]. The “original” ICH score was initially developed as a tool simply to stratify patients with ICH by injury severity and to assist with communication among clinicians [12]. However, over the past 20 years, various scales have been published (including the original ICH score itself [13]) with estimates of how their scores correlate with patient functional outcome and mortality in cohorts of various populations around the world. Clinicians use a wide variety of these clinical grading scales and assortments of clinical variables when formulating subjective impressions of prognosis [14], often with wide variability in opinions regarding individual patients [15].

Because of this variability, and because the majority of deaths among patients with ICH are preceded by high-stakes clinical decisions to limit their life-sustaining treatments or to completely transition to comfort measures only, concerns among clinicians regarding (1) potential self-fulfilling prophecies and (2) potential goal-discordant care when facilitating these decisions are paramount [16–20]. The specific objectives of these Neurocritical Care Society and Deutsche Gesellschaft für Neurointensivmedizin (German Society for Neurointensive and Emergency Medicine) guidelines are to define clinical outcomes important for such goals-of-care decisions regarding patients with ICH and provide evidence-based opinions regarding the formal reliability of some of the most common clinical variables and relevant clinical grading scales for predicting those outcomes—that is, to the degree that an established set value range or definition of one or several of these predictors could solely drive a decision to limit life-sustaining therapy on its/their own. These reliability assessments of individual clinical variables and grading scales are meant to assist clinicians with a better understanding of their most appropriate use when formulating real-world prognostic impressions for patients with ICH and counseling patients and surrogates in the intensive care unit (ICU) about likely outcomes and implications for management.

### Scope, Purpose, and Target Audience

The scope of these Grading of Recommendations Assessment, Development and Evaluation (GRADE) guidelines is the prognostication of neurological outcome in critically ill adult patients with ICH. The purpose of these guidelines is to provide evidence-based recommendations on the reliability of key predictors of neurological outcome in critically ill adult patients with ICH, to aid clinicians in formulating a prognosis. The target audience consists of clinicians responsible for such counseling.

### How to Use These Guidelines

These guidelines provide recommendations on the reliability of select demographic and clinical variables as well as prediction models when counseling patients with ICH and/or their surrogates. We formally categorized these predictors as reliable, moderately reliable, or not reliable. We based this categorization on a GRADE-based assessment of certainty in the body of evidence, as well as effect size (quantification of predictor accuracy) across published studies, as shown in Table 1.

**Table 1:** Reliable and moderately reliable predictors

Category of predictor/ model	GRADE criteria					Point estimates of accuracy in the body of evidence	Use during counseling of patients or surrogates?	Presence of additional specific reliable or moderately reliable predictors required for use during counseling?	Suggested language during counseling of patients or surrogates	
	Risk of bias	Inconsistency	Imprecision	Indirectness	Quality of evidence- overall				Likelihood of outcome	Disclaimer of uncertainty during counseling
	One downgrade permitted	Downgrade NOT permitted	Downgrade NOT permitted	Downgrade NOT permitted	Moderate or high	High	Yes	Preferred, but not absolutely required	“Very likely”	Present, but low
	One downgrade permitted	Downgrade NOT permitted	One downgrade permitted	One downgrade permitted	Any	High	Yes	Yes	“Likely”	Substantial
	Downgrade permitted	Downgrade permitted	Downgrade permitted	Downgrade permitted	Any	Any	No^a^	Not applicable	Not applicable	Not applicable

*GRADE*, Grading of Recommendations Assessment, Development, and Evaluation

^a^Many predictors designated “not reliable” are practically used by clinicians in formulating and communicating real-world subjective impressions of prognosis. The purpose of these guidelines is to identify predictors, if any, that meet reliable or moderately reliable criteria

Reliable predictors, for the purposes of these guidelines, may be used to formulate a prognosis when the appropriate clinical context is present in the absence of potential confounders. These are predictors with clear, actionable thresholds or clinical/radiographic definitions and a low rate of error in prediction of poor outcomes, with at least moderate certainty in the body of evidence. When the prognosis is formulated on the basis of one or more reliable predictors, a clinician may describe the outcome as “very likely” during counseling. Given the inherent limitations in neuroprognostication research, the clinician must nevertheless acknowledge the presence of uncertainty—albeit low—in the prognosis during counseling.

Moderately reliable predictors may be used for prognostication *only* when additional reliable or moderately reliable predictors are present, in addition to the appropriate clinical context. These are also predictors with clear, actionable thresholds or clinical/radiographic definitions and a low rate of error in prediction of poor outcomes, but with lower certainty in the body of evidence, frequently as a result of smaller studies that result in imprecision. When the prognosis is formulated on the basis of multiple moderately reliable predictors, the clinician may describe the outcome as “likely” during counseling but must acknowledge “substantial” uncertainty in the prognosis.

As mentioned, recommendations for reliable or moderately reliable predictors should be able to specify clear, actionable thresholds or clinical/radiographic definitions for clinician action or judgment. Although the panelists recognize that those predictors that do not meet this particular criterion or those other GRADE criteria above are often used by clinicians in formulating their subjective impressions of prognosis, they have nevertheless been deemed not reliable for the purposes of these guidelines and cannot be formally recommended for prognostication on their own. Variables deemed not reliable, however, may be a component of reliable or moderately reliable prediction models.

## Methods

An in-depth description of the methodology used in these guidelines is available in the Supplementary Appendix 1. Per GRADE approach, the Population, Intervention, Comparator, Outcome, Timing, Setting questions were each framed a priori as follows:

“When counseling ICH patients or their surrogates, should <*predictor, with time of assessment if appropriate*> be considered a reliable predictor of <*outcome, with time frame of assessment*>?”

### Selection of Predictors

Candidate predictors were selected based on clinical relevance *and* recent and overall attention in the literature. Individual clinical variables and grading scales were considered “clinically relevant” if, in the opinion of the content experts, they were realistically likely to be used by many clinicians in real-world counseling conversations with patients and surrogates. For individual clinical variables, those available at the time of admission were preferentially selected given the implications of early prognostication on subsequent patient management. A minimum appropriate body of literature was considered present if at least two studies were available using multivariate analysis establishing independent prediction of a clinical outcome or reports discrimination of a predication model.

Based on these criteria, the following candidate predictors were selected:

Clinical variables:

1. Age [21–28]
2. Clinical examination on admission [29]
3. ICH volume on admission [30, 31]
4. Infratentorial location [32]
5. Intraventricular blood on admission [33–41]
6. Anticoagulation at the time of the patient’s ICH onset [42–54]

Clinical grading scales:

1. “Original” ICH score [12, 13]
2. Maximally treated (max-) ICH score [55, 56]

Other individual clinical variables that were considered for formal recommendations in these guidelines up to the point of full-text screening completion included hematoma expansion and neurologic deterioration within 24 h of admission [57–62], preexisting cognitive impairment [63], history of hypertension [64–69], history of diabetes [68, 70–73], and hyperglycemia on admission [73–86]. These were ultimately and pragmatically excluded from the final recommendation listing because either (1) their precise definitions across the available literature were particularly heterogeneous and/or (2) they were thought to be comorbidities applicable to only a select group of patients with ICH, as opposed to being variables that apply to nearly all patients with ICH and that factor into nearly all prognostic discussions.

Multiple ICH clinical grading scales aside from the “original” ICH score and the max-ICH score were also considered for this guideline, up to the completion of full-text literature extraction [12, 13, 55, 56, 87–109]. The panel decided to focus on recommendations on the original ICH score and the max-ICH score for a number of pragmatic reasons. The original ICH score has the most external validation attempts with both discrimination and calibration reported in the literature [11, 110–130]. Although several scales have attempted to account for the self-fulfilling prophecy within their derivation methodology (e.g., the FUNC Score [88]), the max-ICH score has received the most significant recent attention and undergone the most extensive recent external validation among this group [55, 56, 110, 131, 132].

### Selection of Outcomes

Although the prediction of good functional outcome can be important in certain clinical contexts, the ICH outcomes rated by the panel as “critical” for these guidelines, using the GRADE 1–9 scale, were as follows: poor functional outcome assessed at 3 months or later (average rating 9), mortality assessed at 30 days (average rating 9), cognitive status (average rating 8.25), and quality of life (average rating 7.25). Of note, the vast majority of reviewed studies used poor functional outcome/severe disability—defined heterogeneously as a modified Rankin Scale (mRS) of ≥ 3, an mRS of ≥ 4, or a Glasgow Outcome Score ≤ 3—and/or mortality, reported at varying time points, as their main outcomes of interest. Relatively few studies at the level of full-text screening examined cognitive status [133, 134] or quality of life, so the creation of formal recommendations regarding these two outcomes were ultimately deferred.

### Systematic Review Methodology

**Fig. 1:**
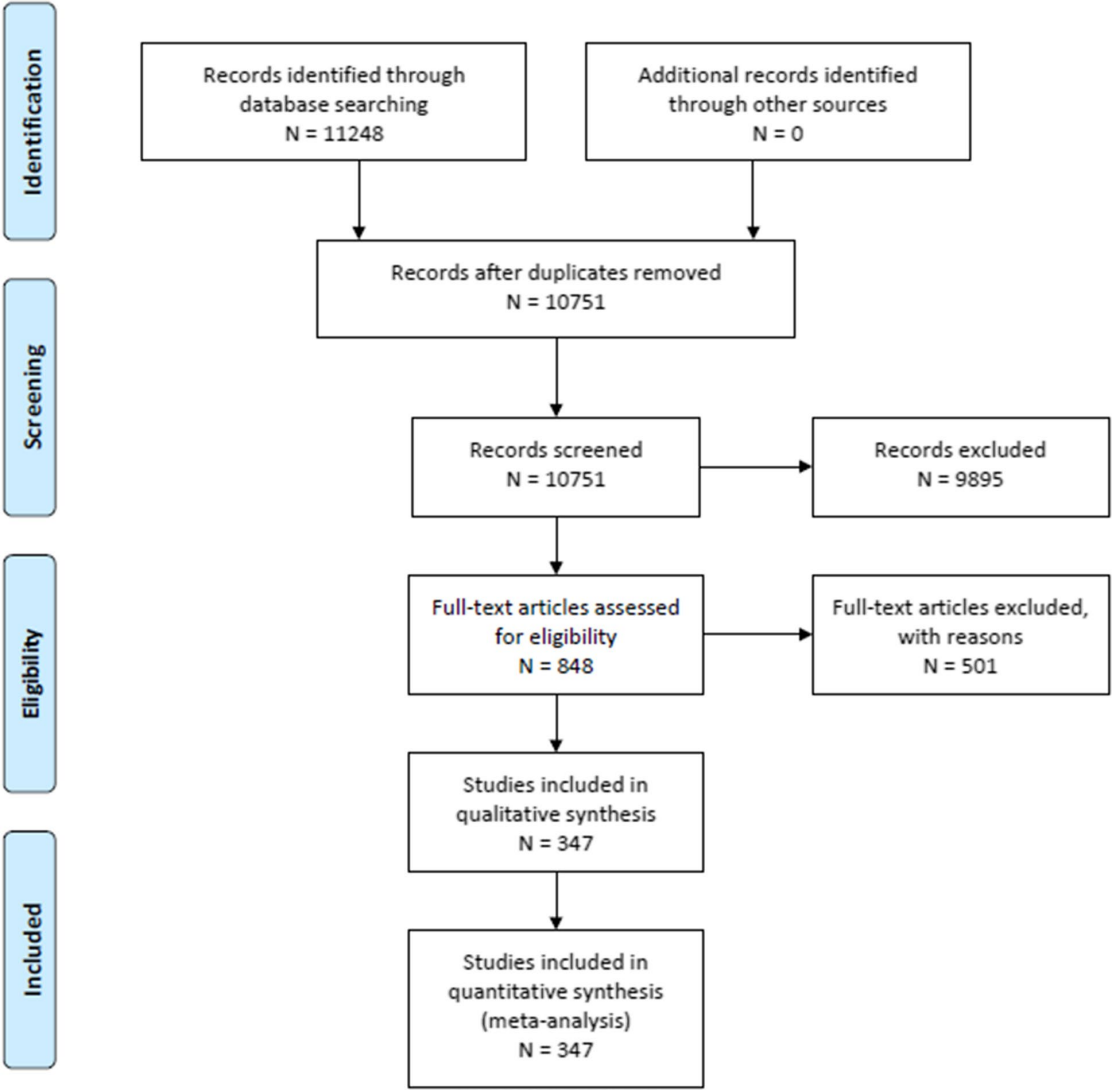
PRISMA 2009 Flow diagram—systematic review: neuroprognostication in intracerebral hemorrhage

An in-depth description of systematic review methodology for these guidelines can be found in Supplementary Appendix 1. Databases searched included MEDLINE via PubMed, EMBASE, Web of Science, and the Cochrane Database of Systematic Reviews. The librarian search string used for this systematic review is in Supplementary Appendix 2, and the PRISMA flow diagram in Fig. 1. Full-text screening was performed with the following exclusion criteria, to select studies—sample size less than 100, mild form of ICH, highly selected subgroup of patients with ICH, studies focused entirely on genetic polymorphism as a predictor, multiple disease states without an adequate sample size and separate analysis in patients with ICH, and interventional studies. Studies were reviewed if they included human study participants, age ≥ 16 years of age; included neuroimaging consistent with contemporary standards used to confirm ICH; included any one of the selected clinical outcomes; evaluated clinically relevant biomarkers on any one of the selected clinical outcomes in two or more published studies; and included at least age and an appropriate measure of disease severity (e.g., clinical examination, ICH volume) as covariates in a multivariate analysis. Studies evaluating clinical grading scales reporting model discrimination were also included. A total of 347 articles out of 10,751 screened articles met our eligibility criteria to guide recommendations.

### Evidence to Recommendation Criteria

1. Quality of evidence/certainty in the evidence and effect size: Predictors described as “reliable” had both a higher overall certainty in the evidence and greater effect size than “moderately reliable” predictors (Table 1). For “reliable” predictors, one downgrade was permitted for risk of bias, but none for inconsistency, imprecision, or indirectness; the overall quality of evidence had to be high or moderate. Single downgrades for risk of bias, imprecision, and indirectness were permitted for “moderately reliable” predictors; but a downgrade for inconsistency was not. Confidence point estimates of accuracy and effect size were required to be relatively high. Clinical prediction models termed “reliable” or “moderately reliable” were required at a minimum to have high discrimination, without evidence of miscalibration. Predictors that did not achieve “reliable” or “moderately reliable” criteria were automatically classified by the panel as “not reliable” from a quality-of-evidence perspective alone.
2. Balance of desirable and undesirable consequences: Prediction of poor outcome in ICH has significant clinical implications when these recommendations are applied in acute clinical settings. Accurate prediction may assist patients and surrogates in discussing goals of care and finding resources early in the course of disease for optimal recovery. However, inaccurate prediction may lead to either false hope or premature limitation of life support and result in overutilization or underutilization of resources, respectively. Both of these sets of consequences of inaccurate prediction are important and consequential. However, while composing these guidelines and considering this balance, the panel was particularly concerned with avoiding a possible increase in the rate of premature limitation of life support for patients with ICH in ICUs, given the finality of and challenges in reversing such decisions once care has been converted to comfort only. Therefore, the threshold for acceptable accuracy was high for reliable and moderately reliable predictors. Consideration of this balance was also influenced by general uncertainty regarding the perspectives of individual patients and families with regard to the meaning of “poor outcome,” as summarized below.
3. Values and preferences: As mentioned earlier, the reviewed literature had itself a heterogeneous approach to defining poor functional outcome, including an mRS of ≥ 3, an mRS of ≥ 4, a Glasgow Outcome Score ≤ 3, and other definitions. Compounding this variability within the evidence, the panel had little confidence in being able to predict a priori the values and preferences of any given patient with ICH or family being counseled, with regard to (1) acceptable level of functional outcome and (2) acceptable difficulty and duration of rehabilitation required to possibly achieve that outcome [135]. This issue of possibly disparate values and preferences among individual patients and families was exacerbated by the aforementioned relative lack of studies examining cognitive and quality-of-life outcomes following ICH, outcomes that were considered by the panel to be critical for many patients and families. These factors related to values and preferences had an overall effect of raising the panel’s subjective threshold for rating any given predictor as reliable or moderately reliable on its own for counseling patients with ICH and their families.
4. Resource use: Resource use for attaining predictor values and data for clinical grading scale components and potentially prolonging life-sustaining ICU treatment for patients with ICH were essentially identical across included studies, including computed tomography scanning for diagnosing and monitoring ICH, use of mechanical ventilation for patients with severe disease, and costs related to ICU care. Although not the driving factor for the creation of these recommendations, it is important to note that costs associated with ICU resource utilization are significantly impacted by patients who are judged to have the possibility of achieving an acceptable outcome over time, whether incorrectly or sometimes even correctly (by those who nevertheless need a long time for recovery). Such situations may result in longer time on a mechanical ventilator, prolonged ICU care and rehabilitation, and adjunctive therapies required for ongoing optimal care.

## Good Practice Statements

A summary of all good practice statements is in Table 2. In accordance with recommendations of the GRADE network, these statements were considered by the panel to be actionable, supported by indirect evidence (when appropriate), and essential to guide the practice of neuroprognostication [136]. The good clinical practice reflected in these statements lacked a meaningful body of direct supporting evidence, typically because of insufficient clinical equipoise, but were considered by the panel to be unequivocally beneficial.

**Table 2:** Summary of recommendations—neuroprognostication following intracerebral hemorrhage

**GOOD PRACTICE STATEMENTS**
Aside from the most clinically devastated patients, neuroprognostication for patients with ICH should in general be deferred for at least the first 48–72 h of ICU admission (conditional recommendation, evidence not graded).
Factors such as preexisting cognitive impairment, poor baseline level of functioning, preexisting illness associated with limited life-expectancy, and multiorgan failure are considered at the time of prognostication. These factors are distinct from the scope of these guidelines (strong recommendation, evidence not graded).
Long term cognitive and psychological impairments are common among patients with ICH who do not necessarily meet criteria for a poor functional outcome. Care should be taken during counseling of individual patients’ families to understand what outcomes would have been most valued by the patient (strong recommendation, evidence not graded).
Patients and surrogates should be counseled that ultimate neurological recovery among patients with ICH may occur over a variable period of time, from several days to several months or years (strong recommendation, evidence not graded).
**PREDICTORS OF POOR FUNCTIONAL OUTCOME AT 3 MONTHS OR LATER**
***Clinical Variables***
**Age**
When counseling patients with ICH or their surrogates, we **suggest** the patient’s **age alone not be considered a reliable predictor** of poor functional outcome assessed at 3 months or later (weak recommendation; very low quality evidence).
**Clinical exam on admission**
When counseling patients with ICH or their surrogates, we **suggest** the patient’s **clinical exam on admission alone not be considered a reliable predictor** of poor functional outcome assessed at 3 months or later (weak recommendation; low quality evidence).
**ICH volume on admission**
When counseling patients with ICH or their surrogates, we **suggest** the patient’s **ICH volume on admission alone not be considered a reliable predictor** of poor functional outcome assessed at 3 months or later (weak recommendation; low quality evidence).
**Infratentorial location**
When counseling patients with ICH or their surrogates, we **suggest** that an **infratentorial location alone of the patient’s ICH not be considered a reliable predictor** of poor functional outcome assessed at 3 months or later (weak recommendation; low quality evidence).
**Intraventricular hemorrhage**
When counseling patients with ICH or their surrogates, we **suggest** that the **presence of intraventricular hemorrhage on admission alone not be considered a reliable predictor** of poor functional outcome assessed at 3 months or later (weak recommendation; low quality evidence).
**Anticoagulation**
When counseling patients with ICH or their surrogates, we **suggest** that **anticoagulation at the time of the patient’s ICH onset alone not be considered a reliable predictor** of poor functional outcome assessed at 3 months or later (weak recommendation; low quality evidence).
***Clinical Grading Scales***
**Original ICH score**
When counseling patients with ICH or their surrogates, we **suggest** that the patient’s **“original” ICH Score not be considered a reliable predictor** of poor functional outcome at 3 months and beyond (weak recommendation; low quality evidence).
**Max-ICH score**
When counseling patients with ICH or their surrogates, we **suggest** that the patient’s **max-ICH Score not be considered a reliable predictor** of poor functional outcome at 3 months and beyond (weak recommendation; low quality evidence).
**PREDICTORS OF 30-DAY MORTALITY**^**a**^
***Clinical Grading Scales***^**b**^
**Original ICH score**
When counseling patients with ICH or their surrogates, we **suggest** that the patient’s **“original” ICH Score not be considered a reliable predictor** of mortality at 30 days (weak recommendation; very low quality evidence).

*ICH* intracerebral hemorrhage, *ICU* intensive care unit

^a^Recommendations for individual clinical variables as predictors of 30-day mortality are summarized in Supplementary Appendix 3. Similar to prediction of functional outcome at 3 months or later, the panel suggested that none of the individual variables were reliable on their own for 30-day mortality prediction

^b^There was not sufficient evidence to generate a formal recommendation on utilizing the max-ICH score for prediction of 30-day mortality

### Good Practice Statement #1

Aside from the most clinically devastated patients, neuroprognostication for patients with ICH should in general be deferred for at least the first 48–72 h of ICU admission (conditional recommendation, evidence not graded).

### Rationale

The “clinically devastated patient” is one who has an immediate threat to life due to a neurologic cause [137]. Otherwise, the 2022 American Heart Association/American Stroke Association Guideline for ICH management recommends postponement of do-not-attempt-resuscitation orders or other limitations of life-sustaining treatment until the at least the second full day of hospitalization [138]. While the optimal amount of time for aggressive treatment for patients with severe ICH is uncertain and is likely not uniform, this panel agreed with the principle of the American Heart Association/American Stroke Association recommendation in its attempt to advise clinicians to be wary of self-fulfilling prophecies and to be aware of the independent association of do-not-resuscitate orders with outcomes of patients with ICH [19].

### Good Practice Statement #2

Factors such as preexisting cognitive impairment, poor baseline level of functioning, preexisting illness associated with limited life-expectancy, and multiorgan failure should be considered at the time of prognostication. These factors are distinct from the scope of these guidelines (strong recommendation, evidence not graded).

### Rationale

Preexisting comorbidities and ICU complications can at times be more of a driving factor for patient’s ultimate functional outcome or ability to survive than perhaps the severity of an ICH itself [8, 139]. While the panel made the aforementioned pragmatic decision to focus the scope of individual clinical variables selected for this guideline mostly to age and key clinical and radiographic factors associated with ICH severity, it affirms the critical importance of other systemic clinical variables in determining patient outcome [96].

### Good Practice Statement #3

Long-term cognitive and psychological impairments are common among patients with ICH who do not necessarily meet criteria for a poor functional outcome. Care should be taken during counseling of individual patients’ families to understand what outcomes would have been most valued by the patient (strong recommendation, evidence not graded).

### Rationale

While this guideline focuses on the outcomes of functional outcome and mortality for reasons outlined earlier, patients who survive ICH may experience cognitive and psychological problems, depending in part on the location and severity of injury [140]. The incidence of cognitive and psychological impairment ranges from 17 to 40% at 3 months and 19–63% at 6–12 months after ICH [133, 134, 141, 142]. Survivors of primary ICH should be evaluated for cognitive and psychological impairments, and appropriate therapies should be discussed among clinical team members and patients/surrogates. Goals of care and progress should be discussed between clinical teams and patient/surrogates to maximize efficacy and minimize adverse events from these therapies.

### Good Practice Statement #4

Patients and surrogates should be counseled that ultimate neurological recovery among patients with ICH may occur over a variable period of time, from several days to several months or years (strong recommendation, evidence not graded).

### Rationale

The appropriate length of observation to determine ultimate recovery—functional, cognitive, and psychological—is uncertain due to heterogeneity in study design among few available studies with long follow-up [2]. Functional recovery may be seen in 50–64% of patients at up to 32 months after ICH, and psychological recovery may take several years [143]. Ultimate neurological recovery may depend on several factors including resource availability, socioeconomical status, and patients’ clinical status. Clinicians and patients/surrogates should discuss goals of care and progress periodically to set reasonable expectations.

## Recommendations: Clinical Variables as Predictors

### Outcome: Poor Functional Outcome at 3 Months or Later

The recommendations for individual clinical variable prediction of poor functional outcome at 3 months or later are summarized in Table 2, and the accompanying GRADE Evidence Profile and Summary of Findings table are contained in Table 3.

**Table 3:** GRADE evidence profile/summary of findings table: neuroprognostication—intracerebral hemorrhage

Outcome	Variable	Quality of evidence							Supplementary Appendix 1
		RoB	Inconsistency	Indirectness	Imprecision	Publication Bias	Reasons to Upgrade	QoE-summary (high/moderate/low/very low)	
Individual clinical variables									
90-day functional outcome	Age	↓ Self-fulfilling prophecy bias	↓ Inconsistent results amongst studies		↓ Small, monocentric sample sizes for most prospective studies			Very low	Split amongst studies – multiple studies with ORs around 1.0. Other studies with ORs significantly higher, for older ages and poor outcome
90-day functional outcome	Clinical exam on admission	↓ Self-fulfilling prophecy bias			↓ Small, monocentric sample sizes for most prospective studies			Low	ORs around 0.7 or 1.3, depending on how clinical exam and outcome are defined
90-day functional outcome	ICH volume on admission (for supratentorial ICH)	↓ Self-fulfilling prophecy bias			↓ Small, monocentric sample sizes for most prospective studies			Low	Most studies with ORs around 1.0. Some studies that split volume into categorical groups had higher ORs for poor outcome in larger volume groups
Clinical grading scales									
90-day functional outcome	Infratentorial location	↓ Self-fulfilling prophecy bias			↓ Small, monocentric sample sizes for most prospective studies; uncertainty of effect size			Low	ORs consistently above 1 for poor outcome, but magnitude of effect with very wide range (2.0 to > 10.0)
90-day functional outcome	Intraventricular blood on admission	↓ Self-fulfilling prophecy bias			↓ Small, monocentric sample sizes for most prospective studies; uncertainty of effect size			Low	Reported ORs are wide-ranging, from around 1.0 all the way up to 15.0 depending on how IVH is defined
90-day functional outcome	Anticoagulation at the time of the patient’s ICH onset	↓ Self-fulfilling prophecy bias			↓ Small, monocentric sample sizes for most prospective studies; uncertainty of effect size			Low	ORs for several studies hovering around 1.0
90-day functional outcome	Original ICH Score	↓ Self-fulfilling prophecy bias, few studies report calibration			↓ Small, monocentric sample sizes for most prospective validation studies			Low	AUC 0.67–0.85; has not been shown to be more accurate than subjective provider judgment
90-day functional outcome	Max ICH Score	↓ Possibility that analysis of only maximally treated group may bias outcomes positively			↓ Limited worldwide validation at this point			Low	AUC 0.8–0.86
30-day mortality	Original ICH Score	↓ Self-fulfilling prophecy bias, few studies report calibration	↓ Predicted mortality rates highly variable depending on ECL in cohort		↓ Small, monocentric sample sizes for most prospective validation studies			Very low	AUC 0.72–0.89

The GRADE evidence profile (EP) and summary of findings (SoF) table for individual clinical variable prediction of 30-day mortality is summarized in Supplementary Appendix 4

There was not sufficient evidence to generate a GRADE EP and SoF table FOR the max-ICH score with respect to predicting 30-day mortality

1. Question: When counseling patients with ICH or their surrogates, should age alone be considered a reliable predictor of poor functional outcome assessed at 3 months or later?
  Description of the predictor: Older age may be a surrogate for baseline infirmity, comorbidities, and/or and diminished cerebral reserve [144]. There are few ICH prognostication studies examining age as the primary predictor of interest [21–28], but it is a ubiquitous covariate in studies of nearly all potential predictors, as well as a component variable incorporated in nearly every ICH clinical grading scale. Most ICH studies incorporate age as a continuous variable. Those that either dichotomize or stratify it do not use standardized categorical cutoffs.
  Recommendation: When counseling patients with ICH or their surrogates, we suggest the patient’s age alone not be considered a reliable predictor of poor functional outcome assessed at 3 months or later (weak recommendation; very low quality evidence).
  Rationale: Older age was independently associated with poor ICH outcome in the majority of the reviewed studies of other predictors, and the panel acknowledged that age is likely factored into many clinicians’ subjective assessments of predicted functional outcomes for their patients with ICH. However, the quality of evidence for focusing on age *alone* as a potentially reliable predictor of functional outcome was downgraded over multiple concerns from the panel. These concerns included (1) risk of bias from self-fulfilling prophecies regarding limitations of life-sustaining therapy decisions made within study cohorts; (2) inconsistency regarding reported effect sizes, or the magnitude of associations of age with poor functional outcome; and (3) possible imprecision. A decision to suggest age alone as not reliable was also influenced by lack of consensus over the optimal cutoff values for defining “older” age groups and significant uncertainty regarding a consensus definition of “poor functional outcome,” as perceived by patients with ICH and their surrogates with varying values and preferences. Furthermore, the stakes of an inaccurate prediction of functional outcome—i.e., potential limitation of life-sustaining therapy for a patient who could have eventually achieved a perceived favorable outcome—raised the threshold among panel members for suggesting age alone as having sufficiently precise evidence to be considered as potentially reliable for neuroprognostication discussions.
2. Question: When counseling patients with ICH or their surrogates, should clinical examination on admission alone be considered a reliable predictor of poor functional outcome assessed at 3 months or later?
  Description of the predictor: The clinical examination on admission for patients with ICH is a marker of initial severity of injury [29]. Similar to age, the admission clinical examination is a ubiquitous covariate in studies of nearly all potential predictors of ICH outcome, as well as a component variable incorporated in nearly every clinical grading scale. Of note, the methodology for defining a patient’s clinical examination varies significantly in the ICH literature, with many studies using various stratifications of the Glasgow Coma Scale (GCS) and others using various categorizations based on the National Institutes of Health Stroke Scale (NIHSS) [9].
  Recommendation: When counseling patients with ICH or their surrogates, we suggest the patient’s clinical examination on admission alone not be considered a reliable predictor of poor functional outcome assessed at 3 months or later (weak recommendation; low quality evidence).
  Rationale: Similar to older age, a poor clinical examination on admission was independently associated, to some extent, with poor ICH outcome in the majority of the reviewed studies of other predictors. We acknowledge that clinical examination is likely factored into many clinicians’ early subjective assessments of predicted functional outcomes for their patients with ICH. However, the quality of evidence for focusing on admission clinical examination *alone* as a potentially reliable predictor of functional outcome was downgraded over (1) risk of bias from self-fulfilling prophecies and (2) possible imprecision. Furthermore, (1) varying methods used in studies to score the clinical examination, (2) lack of consensus over the optimal cutoff values for categorizing these scores, and (3) significant uncertainty regarding a consensus definition of “poor functional outcome,” as perceived by patients with ICH and their surrogates, all contributed to this recommendation. Once again, the stakes of an inaccurate prediction of functional outcome and premature limitation of life-sustaining therapy raised the threshold for comfort in suggesting admission clinical examination as having sufficiently precise evidence to be considered as potentially reliable for neuroprognostication discussions, by itself.
3. Question: When counseling patients with ICH or their surrogates, should ICH volume on admission alone be considered a reliable predictor of poor functional outcome assessed at 3 months or later?
  Description of the predictor: ICH volume on admission is an intuitive radiographic measurement of severity of injury [30, 31]. Similar to age and clinical examination, ICH volume is also a ubiquitous covariate in studies of nearly all potential predictors of ICH outcome. Studies that report ICH volume use various cutoffs for categorization, with some having different cutoffs within the same study for patients with different ICH locations (e.g., supratentorial vs. infratentorial) [9].
  Recommendation: When counseling patients with ICH or their surrogates, we suggest the patient’s ICH volume on admission alone not be considered a reliable predictor of poor functional outcome assessed at 3 months or later (weak recommendation; low quality evidence).
  Rationale: Similar to age and admission clinical examination, a large ICH volume on admission was independently associated to some extent with poor ICH outcome in many reviewed studies of other predictors. Those studies with larger cutoff volumes for defining their “large” categories were able to demonstrate strong associations with poor functional outcome. We acknowledge that ICH volume, like age and clinical examination, is likely factored into many clinicians’ early subjective assessments of predicted functional outcomes for their patients with ICH. However, similar to aforementioned variables, the quality of evidence for focusing on ICH volume *alone* as a potentially reliable predictor of functional outcome was downgraded over (1) risk of bias from self-fulfilling prophecies and (2) possible imprecision. The varying cutoff values among reviewed studies for categorizing small versus large volume hemorrhages, the influence of location on the impact of the volume of an ICH, and significant uncertainty regarding a consensus definition of “poor functional outcome,” as perceived by patients with ICH and their surrogates, were all factored into recommendation development. Similar to age and admission clinical examination, the stakes of an inaccurate prediction of functional outcome and premature limitation of life-sustaining therapy raised the panel’s threshold for comfort in suggesting admission ICH volume alone as having sufficiently precise evidence to be considered as potentially reliable for neuroprognostication discussions.
4. Question: When counseling patients with ICH or their surrogates, should an infratentorial location alone be considered a reliable predictor of poor functional outcome assessed at 3 months or later?
  Description of the predictor: Because of the critical functions of the brainstem in maintaining a patient’s independent functional status, as well as the threat to the brainstem that a cerebellar ICH with sufficient size and/or accompanying cerebral edema can pose, an ICH located inferior to the cerebellar tentorium can often be of particular concern in discussions of prognosis. Most studies of ICH prognostic factors do not define ICH location in more detail aside from this supratentorial versus infratentorial distinction [9].
  Recommendation: When counseling patients with ICH or their surrogates, we suggest that an infratentorial location alone of the patient’s ICH not be considered a reliable predictor of poor functional outcome assessed at 3 months or later (weak recommendation; low quality evidence).
  Rationale: Similar to previously discussed variables, infratentorial ICH location was independently associated with poor ICH outcome in a number of studies. However, the quality of evidence for focusing on infratentorial ICH location *alone* as a potentially reliable predictor of functional outcome was downgraded over (1) risk of bias from self-fulfilling prophecies and (2) possible imprecision, with wide confidence intervals reported among studies regarding the degree of association between infratentorial location and poor outcome. Also, varying functional neuroanatomy among specific infratentorial locations (e.g., reticular activating system vs. cerebellar hemisphere), the influence of ICH volume on the impact of ICH location, and the recurring uncertainty regarding a consensus definition of poor outcome itself factored into recommendation writing. With regards to a balance of desirable and undesirable consequences, the concern for premature limitation of life-sustaining therapy once again raised the panel’s threshold for suggesting infratentorial ICH volume as having sufficiently precise evidence to be considered as potentially reliable for neuroprognostication discussions, by itself.
5. Question: When counseling patients with ICH or their surrogates, should the presence of intraventricular hemorrhage (IVH) on admission alone be considered a reliable predictor of poor functional outcome assessed at 3 months or later?
  Description of the predictor: IVH, either on its own or in conjunction with ICH, can disrupt normal cerebrospinal fluid (CSF) dynamics and lead to hydrocephalus. Hydrocephalus that is not sufficiently treated may result in mass effect on critical brain structures. Hydrocephalus that is treated with attempted CSF diversion and potentially intraventricular tissue plasminogen activator via an extraventricular drain may still lead to future morbidity and mortality and/or require permanent intracranial shunt placement by neurosurgical teams [145]. Despite the impact of the volume and location of IVH on the degree of CSF flow disruption, IVH is simply reported as present or absent in many studies of ICH prognosis [9].
  Recommendation: When counseling patients with ICH or their surrogates, we suggest that the presence of IVH on admission alone not be considered a reliable predictor of poor functional outcome assessed at 3 months or later (weak recommendation; low quality evidence).
  Rationale: Historically, the presence of IVH, reported as a purely binary variable, has been independently associated with poor ICH outcome [33–41]. The panel downgraded the quality of evidence for focusing on the presence of IVH *alone* as a potentially reliable predictor of functional outcome due to (1) risk of bias from self-fulfilling prophecies and (2) possible imprecision. Concerns were expressed regarding the lack of information in many studies regarding the degree of IVH among their cohorts, lack of information in the reviewed studies regarding the aggressiveness of treatment of IVH, recurring uncertainty regarding a consensus definition of poor outcome itself, and the stakes that the threat of even occasional premature limitation of life-sustaining therapy raise.
6. Question: When counseling patients with ICH or their surrogates, should anticoagulation at the time of the patient’s ICH onset alone be considered a reliable predictor of poor functional outcome assessed at 3 months or later?
  Description of the predictor: Anticoagulation at the time of ICH onset is a strong risk factor for ICH expansion and neurologic deterioration [42–54]. The mainstay of treatment for anticoagulated patients is to reverse the coagulopathy emergently. Even anticoagulated patients who are reversed emergently face additional clinical challenges, including assessment of continued adequate reversal and decisions involving risks and benefits of restarting anticoagulation, given their original indications for the patient requiring anticoagulation [146].
  Recommendation: When counseling patients with ICH or their surrogates, we suggest that anticoagulation at the time of the patient’s ICH onset alone not be considered a reliable predictor of poor functional outcome assessed at 3 months or later (weak recommendation; low quality evidence).
  Rationale: While many studies have shown a strong independent association between anticoagulation at time of ICH onset and poor functional outcome, the panel downgraded the quality of evidence for focusing on the anticoagulation *alone* as a potentially reliable predictor. This decision was due to risk of bias from self-fulfilling prophecies; possible imprecision; general lack of information in reviewed studies regarding the speed, method, and success of ICH reversal; general lack of information regarding the types of initial anticoagulation that patients were receiving [46]; recurring uncertainty regarding a consensus definition of poor outcome itself; and, once again, the stakes raised by even the occasional threat of premature limitation of life-sustaining therapy.

### Outcome: Mortality

While the panel did generate recommendations for clinical variables and their use in prognostication of 30-day mortality, the discussion of the quality of evidence for the prediction of 30-day mortality among clinical variables was thought not only to mirror that for the prediction of functional outcome, but also to be further downgraded by the potential high risk of bias from the self-fulfilling prophecy [147]. A summary of recommendations and accompanying GRADE Evidence Profile and Summary of Findings table for individual clinical variable prediction of 30-day mortality are in Supplementary Appendix 3. Similar to the recommendations for functional outcome, the panel suggested that none of the selected individual clinical variables were reliable on their own for 30-day mortality prediction.

## Recommendations: Clinical Grading Scales

The recommendations for (1) individual clinical grading scales as predictors of poor functional outcome at 3 months or later and (2) the original ICH score as a predictor of 30-day mortality are summarized in Table 2, and the accompanying GRADE Evidence Profile and Summary of Findings table are contained in Table 3. For mortality particularly at the 30-day time point, a formal recommendation for the max-ICH score was deferred based on lack of literature.

### Outcome: Functional Outcome

1. Question: When counseling family members or surrogates of patients suffering from spontaneous intracerebral hemorrhage, should the “original” ICH score be considered a reliable predictor of functional outcome assessed at three months or later?
  Description of the prediction model: The original ICH score was initially developed from a bicentric cohort of 152 North American patients with nontraumatic ICH [12]. The original ICH score includes five characteristic factors determined to independent predictors of outcome easily and rapidly determined at time of ICH presentation. GCS score, most strongly associated with outcome, was divided in different groups: GCS score 13 – 15 = 0 points, GCS score 5 – 12 = 1 point, GCS score 3 – 4 = 2 points. Further components are ICH volume, cm^3^ (≥30; <30); intraventricular extension of hemorrhage (yes/no); infratentorial origin (yes/no); and age, years (≥80; <80). ICH volume ≥ 30 cm^3^, intraventricular extension of hemorrhage, infratentorial hemorrhage, and age ≥ 80 years are each assigned 1 point. Calculation of the original ICH score gives a sum of maximal 6 points. The validation for functional outcome (3, 6, and 12 months) was provided in a further prospective study of 243 patients with nontraumatic ICH demonstrating a higher likelihood of unfavorable outcome with increasing original ICH score [13]. Numerous studies have performed external validation of the original ICH score in prospective monocentric and multicentric cohorts [11, 110–130], including data analysis of good discrimination (area under the curve (AUC), range from 0.67–0.89) for mortality and unfavorable outcome up to 12-month follow-up.
  Recommendation: When counseling patients with ICH or their surrogates, we suggest that the patient’s “original” ICH score not be considered a reliable predictor of poor functional outcome at 3 months or later (weak recommendation; low quality evidence).
  Rationale: The body of evidence for the original ICH score as a predictor of functional outcome was downgraded over concerns regarding (1) possible risk of bias from the self-fulfilling prophecy; and (2) possible imprecision, given its derivation and prospective observational validation studies are mostly monocentric with variable sample sizes. Furthermore, one study has found that the “original” ICH score appears to be outperformed by the early subjective judgment of clinicians with regards to 3-month functional outcome prediction [148]. In addition, the decision to suggest this score as not reliable for real-world prognostication of patients was also influenced by uncertainty regarding values and preferences among individual patients with ICH and/or their surrogates with respect to acceptable outcome.
2. Question: When counseling family members or surrogates of patients suffering from spontaneous intracerebral hemorrhage, should the max-ICH score be considered a reliable predictor of functional outcome assessed at three months or later?
  Description of the prediction model: The max-ICH score was developed to provide severity assessment for functional long-term outcome with minimized confounding by care limitations among 583 patients from a prospective monocentric German registry [55]. The cohort included 112 patients with ICH with early care limitations and 471 with maximal treatment. The max-ICH score focuses on six components which sum up to a maximum of 10 points. Included variables are NIHSS at hospital admission (0–6 = 0 points, 7–13 = 1 point, 14–20 = 2 points, ≥ 21: 3 points); age, years (≥ 69 = 0 points, 70–74 = 1 point, 75–79 = 2 points, ≥80: 3 points); IVH (yes = 1 point); oral anticoagulation (yes = 1 point); lobar ICH volume, cm^3^ (≥ 30 = 1 point); and nonlobar ICH volume, cm^3^ (≥ 10 = 1 point). All imaging components indicate measures on initial computed tomography examinations. External validation has been completed in several cohorts [56, 110, 132], with good discrimination and calibration for functional outcome demonstrated.
  Recommendation: When counseling patients with ICH or their surrogates, we suggest that the patient’s max-ICH score not be considered a reliable predictor of poor functional outcome at 3 months or later (weak recommendation; low quality evidence).
  Rationale: Quality of evidence for the max-ICH score was downgraded over concerns regarding (1) possible risk of bias, in that analysis of only maximally treated patients with ICH may in turn bias outcomes positively; and (2) relatively limited worldwide validation at this point, in comparison to the original ICH score. Furthermore, results of head-to-head comparisons of the discrimination of the max-ICH score to that the original ICH score for long-term functional outcome prediction at this point have been mixed [110]. No studies have examined either the accuracy of the max-ICH score compared with subjective clinician judgment for 3-month functional outcome prediction. Also, the decision to suggest the max-ICH score as not reliable for real-world prognostication of patients with respect to functional outcome was once again also influenced by uncertainty regarding values and preferences among individual patients with ICH and/or their surrogates with respect to acceptable outcome.

### Outcome: Mortality

Question: When counseling family members or surrogates of patients suffering from spontaneous ICH, should the “original” ICH score considered a reliable predictor of mortality at 30 days?

Recommendation: When patients with ICH or their surrogates, we suggest that the patient’s “original” ICH score not be considered a reliable predictor of mortality at 30 days (weak recommendation; very low quality evidence).

Rationale: Quality of evidence for the original ICH score was downgraded over concerns regarding (1) possible risk of bias, mostly from the self-fulfilling prophecy [147]; and (2) possible imprecision, given its derivation and prospective observational validation studies are mostly monocentric with variable sample sizes. For predicting 30-day mortality, quality of evidence for the original ICH score was further downgraded for (3) possible inconsistency, given the variability of mortality rates among various levels of the score observed in different studies and cohorts [147]. In addition, the decision to suggest the original ICH score as not reliable for real-world prognostication of patients was also influenced by uncertainty regarding values and preferences among individual patients with ICH and/or their surrogates with respect to acceptable outcome.

A detailed table specifying the quality-of-evidence judgment of each relevant individual study included at the full-text screening level is available in Supplementary Appendix 4.

## Future Directions

Despite a very large and ever-growing amount of literature on independent predictors of ICH functional outcome and mortality [3, 8] and a large number of published clinical grading scales [10], this panel did not judge any single clinical variable or scale alone to meet criteria for reliability or moderate reliability in real-world counseling of patients with ICH and families about prognosis. This conclusion was not surprising for a number of reasons, some of which are inherent in the construction of the Population, Intervention, Comparator, Outcome, Timing, Setting questions, and some of which are inherent to the fundamental uncertainty of neuroprognostication. However, the overriding consideration that drove recommendations was concern about inappropriate early limitation of care on the basis of predictors with insufficient accuracy, as discussed in the “Evidence to recommendation” section (“balance of desirable and undesirable consequences”). This risk is well documented in patients with ICH [16, 149]. The self-fulfilling prophecy remains a difficult source of bias to control for in cohort studies of all types of severe acute brain injury. This fact is especially true with regards to mortality as the outcome of interest. However, even with regards to predicting functional outcomes, attempts to develop grading scales based on cohorts excluding patients with care limitations are helpful but can raise questions of optimistic biases in their projections [150]. Studies of patient cohorts in which care limitations are simply not allowed are unethical.

Additionally, recommendations regarding individual predictor variables should ideally be written with clear, actionable thresholds or clinical/radiographic definitions. This task is more straightforward in some disease states in which certain key variables lend themselves to standardized definitions (e.g., present vs. absent bilateral pupillary reflex for patients with hypoxic-ischemic injury), but much less so for ICH, where the methods and cutoffs for defining variables such as the “clinical examination on admission” have not been standardized. Predictor variables and clinical grading scales described in this guideline may have qualitative value in generating an overall prognostic impression, but all lack formal reliability for quantitatively defining specific point estimates for accurate and precise numeric prognostication that would be actionable. For example, in general having a larger hematoma or a higher max-ICH score is more likely to be associated with a worse prognosis, but specific numeric cut points for precise outcome prediction are not reliable.

Third, aside from an evaluation of the quality of the available evidence, GRADE recommendations also must be based on a panel’s assessment of the values and preferences of patients and families. This panel had very little confidence in being able to predict a priori the values and preferences of any given patient with ICH or family being counseled, with regards to acceptable functional outcome and acceptable difficulty and duration of the “road to recovery” required to possibly achieve that outcome [135].

Despite these barriers in identifying “reliable” predictors of ICH outcome as the word is strictly defined in this guideline, a number of different perspectives have nevertheless been offered in the literature with regards to research that might improve a multimodal approach to ICH prognostication [151], including:

1. Additional external validation of existing major clinical grading scales [131], in particular those that have attempted to adjust for the self-fulfilling prophecy [150];
2. Comparisons of the accuracy of existing clinical grading scales against subjective clinician judgment [148];
3. Incorporation of frailty assessment [152] and/or newer imaging, fluid, or electrophysiology biomarkers [138] into novel grading scales;
4. Incorporation of patient-reported outcome measures into novel grading scales [153];
5. Additional research regarding the optimal timing of prognostic assessment and the quality of post-acute rehabilitation services as a key predictor of interest [154];
6. Application of machine learning techniques for defining thresholds of interest for key clinical variables [155] and developing new predictive models [156, 157];
7. Development of new clinical scales based on cohort of patients undergoing newer techniques for ICH evacuation [158].

## Conclusions

These guidelines provide recommendations on the formal reliability of predictors of poor outcome in the context of counseling patients with ICH and their surrogates. No predictor, by itself, was considered reliable or moderately reliable based on the available body of evidence. Although grading scales may help with clinical communication, general patient risk stratification, clinical trial participant selection, and quality measurement initiatives, clinicians who nevertheless are tasked with formulating their own subjective judgments of poor prognosis and appropriateness of care limitations for their patients with ICH should avoid anchoring those judgments solely on any one clinical variable or published scale.

What research that does exist regarding how clinicians may best reason through their subjective judgments of prognosis for patients with ICH has suggested that (1) such judgments, for all of their potential pitfalls, generally outperform clinical grading scales when compared head-to-head regarding accuracy for long-term outcome and (2) are not necessarily more accurate when focused on a patient’s clinical examination findings versus his or her neuroimaging findings—or even versus his or her social support situation [14]. In our practice, we simply favor a subjective approach guided by both serial clinical examinations and careful assessment of all available neuroimaging and other data, keeping in mind (1) the nonreliability of any one predictor variable or grading scale in isolation and (2) the good practice statements in this guideline when constructing opinions regarding ranges and likelihood of possible outcomes; and communicating those opinions to patients, families, and colleagues.

## Endorsements

These guidelines were endorsed by the American Heart Association, the Deutsche Gesellschaft für Neurochirurgie, and the Society of Critical Care Medicine. The American Academy of Neurology and the American Association of Neurological Surgeons/Congress of Neurological Surgeons affirm the educational value of this document.

### Supplementary Information

Below is the link to the electronic supplementary material.

Supplementary file1 (DOCX 41 KB)

Supplementary file2 (DOCX 32 KB)

Supplementary file3 (DOCX 32 KB)

Supplementary file4 (XLSX 27 KB)

Supplementary file5 (XLSX 347 KB)
