# Supplementary material for: Guidelines for Neuroprognostication in Critically Ill Adults with Intracerebral Hemorrhage
Source: Neurocrit Care. 2023 Nov 3;40(2):395–414. doi: 10.1007/s12028-023-01854-7 (PMC10959839; doi:10.1007/s12028-023-01854-7)
Supplement: Supplementary file 2 — Supplementary file2 (DOCX 32 KB) [file 12028_2023_1854_MOESM2_ESM.docx]

**GRADE summary: Neurocritical Care Society (NCS)/ German Society for Neuro-Intensive and Emergency Medicine (DGNI) guidelines for neuroprognostication: Intracerebral Hemorrhage**

**Supplementary Appendix 2**

**Librarian search string**

Database: All Ovid Medline <1946 - present>

Search Strategy:

--------------------------------------------------------------------------------

1. Acute intracerebral hemorrhage.ti,ab. (446)
2. Acute intracerebral haemorrhage.ti,ab. (67)
3. ICH.ti. (368)
4. acute intracerebral bleed*.ti,ab. (5)
5. spontaneous intracerebral hemorrhage.ti,ab. (939)
6. spontaneous intracerebral haemorrhage.ti,ab. (190)
7. hemorrhagic stroke.ti,ab. (4003)
8. haemorrhagic stroke.ti,ab. (927)
9. intraventricular hemorrhage.ti,ab. (5106)
10. intraventricular haemorrhage.ti,ab. (1226)
11. exp Cerebral Hemorrhage/ (32194)
12. (brain hemorrhage* adj5 cerebral).ti,ab. (33)
13. acute disease/ (206495)
14. acute.ti,ab. (1091522)
15. 13 or 14 (1132385)
16. or/3-12 (40173)
17. 15 and 16 (6876)
18. 1 or 2 or 17 (6974)
19. exp Analysis of Variance/ [includes Multivariate Analysis] (326771)
20. Multivariate.tw. (293720)
21. treatment outcome/ (885811)
22. outcome.tw. (887975)
23. mortality/ or mo.fs. (563611)
24. "Predictive Value of Tests"/ (188131)
25. Disease Progression/ (146077)
26. prediction.tw. (213326)
27. prognostic*.tw. (268771)
28. prognos*.tw. (536821)
29. exp Quality of Life/ (171899)
30. "Quality of life".tw. (241815)
31. scale.ti,ab. (629667)
32. score.ti,ab. (478944)
33. scoring tool*.tw. (605)
34. disability evaluation/ (44760)
35. Survival/ (4559)
36. mortality/ or mo.fs. or death/ (579364)
37. Time factors/ (1143362)
38. 19 or 21 or 22 or 23 or 24 or 26 or 27 or 28 or 29 or 31 or 32 or 33 or 34 or 35 or 37 (4506153)
39. 18 and 38 (3614)
40. prognosis.sh. or diagnosed.tw. or cohort:.mp. or predictor:.tw. or death.tw. or exp models, statistical/ [validated hedge from the Health Information Research Unit, McMaster University] (2446889)
41. 18 and 40 (2422)
42. 39 or 41 (4196)
43. exp cohort studies/ [includes: follow-up studies/, longitudinal studies/, prospective studies/, retrospective studies/, controlled before-after studies/, cross-sectional studies/, or historically controlled study/] (1824723)
44. (Follow-up or longitudinal or prospective or retrospective or before-after or cross-sectional or controlled).tw. (2597128)
45. (predict* or predictor or prognos* or prognost*).ti. (415631)
46. or/43-45 (3640205)
47. 42 and 46 (2268)
48. animals/ not (humans/ and animals/) (4515460)
49. pediatric*.ti. (131199)
50. limit 47 to ("newborn infant (birth to 1 month)" or "infant (1 to 23 months)" or "preschool child (2 to 5 years)" or "child (6 to 12 years)") (247)
51. limit 50 to (address or autobiography or bibliography or biography or case reports) (20)
52. letter.pt. (1015878)
53. 48 or 49 or 50 or 51 or 52 (5623083)
54. 47 not 52 (2259)
55. remove duplicates from 54 (2249)
