# Supplementary material for: Guidelines for Neuroprognostication in Critically Ill Adults with Intracerebral Hemorrhage
Source: Neurocrit Care. 2023 Nov 3;40(2):395–414. doi: 10.1007/s12028-023-01854-7 (PMC10959839; doi:10.1007/s12028-023-01854-7)
Supplement: Supplementary file 3 — Supplementary file3 (DOCX 32 KB) [file 12028_2023_1854_MOESM3_ESM.docx]

**Supplementary Appendix 3**

**Recommendation table for Intracerebral Hemorrhage: Individual clinical variables as predictors of 30-day mortality**

| **VARIABLE** | **TIME OF ASSESSMENT** | **RELIABILITY** |
| --- | --- | --- |
| Age | On admission | Not reliable |
| Clinical exam | On admission | Not reliable |
| ICH volume | On admission | Not reliable |
| Infratentorial location | On admission | Not reliable |
| Intraventricular hemorrhage | On admission | Not reliable |
| Anticoagulation | At time of ICH onset | Not reliable |

**GRADE Evidence Profile/ Summary of Findings table: Neuroprognostication - Intracerebral Hemorrhage**

**Individual clinical variables as predictors of 30-day mortality**

| **Outcome** | **Variable** | **Quality of Evidence** | | | | | | | **Summary of Findings (Narrative of effect size)** |
| --- | --- | --- | --- | --- | --- | --- | --- | --- | --- |
|  |  | **RoB** | **Inconsistency** | **Indirectness** | **Imprecision** | **Publication Bias** | **Reasons to Upgrade** | **QoE- Summary**  **(High/ Moderate/ Low/ Very Low)** |  |
| 30-day mortality | Age | ↓  Self-fulfilling prophecy bias | ↓  Inconsistent results amongst studies |  | ↓  Small, monocentric sample sizes for most prospective studies |  |  | Very low | Split amongst studies – multiple studies with ORs around 1.0. Other studies with ORs significantly higher, for older ages. |
| 30-day mortality | Clinical exam on admission | ↓  Self-fulfilling prophecy bias |  |  | ↓  Small, monocentric sample sizes for most prospective studies |  |  | Low | ORs around 0.7 or 1.3, depending on how clinical exam is defined. |
| 30-day mortality | ICH volume on admission (for supratentorial ICH) | ↓  Self-fulfilling prophecy bias |  |  | ↓  Small, monocentric sample sizes for most prospective studies |  |  | Low | Most studies with ORs around 1.0. Some studies that split volume into categorical groups had higher ORs in larger volume groups. |
| 30-day mortality | Infratentorial location | ↓  Self-fulfilling prophecy bias |  |  | ↓  Small, monocentric sample sizes for most prospective studies; uncertainty of effect size |  |  | Low | ORs consistently above 1.0, but magnitude of effect with very wide range (2.0 to > 10.0). |
| 30-day mortality | Intraventricular blood on admission | ↓  Self-fulfilling prophecy bias |  |  | ↓  Small, monocentric sample sizes for most prospective studies; uncertainty of effect size |  |  | Low | Reported ORs ranging from 2.0-7.0, depending on how IVH is defined and whether hydrocephalus is included as part of definition. |
| 30-day mortality | Anticoagulation at the time of the patient’s ICH onset | ↓  Self-fulfilling prophecy bias |  |  | ↓  Small, monocentric sample sizes for most prospective studies; uncertainty of effect size |  |  | Low | Reported ORs ranging from 1.0 to 2.7; some studies with large confidence intervals. |
